# Rapid Delivery without Previous Pains

**Published:** 1846-06

**Authors:** 


8. *Rapid Delivery without Previous Pains.*—Mr. J. B. Prowse in a letter to the "Lancet," relates the following case, as having an obvious bearing on the question of infanticide. "When a pupil, I was engaged by a poor woman to attend her during her accouchment; she was a native of Ireland, and a remarkably fine and well formed person. She had already borne two children. On the day of her delivery I was requested to call on her, for she thought her confinement was near at hand. Her attendants said she was in no pain, but that she appeared uneasy. I waited on her, and found her on the bed, smiling, and expressing a hope that she had not summoned me unnecessarily; but that as she never suffered much in labor, I would excuse her if she was wrong. On examination I was surprised to find the head of the child in the upper part of the vagina, and was puzzled to account for there having been no pains to lead to a suspicion of the real nature of the case. No sooner was my hand withdrawn, and my back turned to speak to the attendants, than there occured one single effort of the uterus, and the child was in the world."—*Ibid.*

